# High levels of periostin correlate with increased fracture rate, diffuse MRI pattern, abnormal bone remodeling and advanced disease stage in patients with newly diagnosed symptomatic multiple myeloma

**DOI:** 10.1038/bcj.2016.90

**Published:** 2016-10-07

**Authors:** E Terpos, D Christoulas, E Kastritis, T Bagratuni, M Gavriatopoulou, M Roussou, A Papatheodorou, E Eleutherakis-Papaiakovou, N Kanellias, C Liakou, I Panagiotidis, M Migkou, P Kokkoris, L A Moulopoulos, M A Dimopoulos

**Affiliations:** 1 Department of Clinical Therapeutics, National and Kapodistrian University of Athens, School of Medicine, Athens, Greece; 2 Department of Medical Research, 251 General Air-Force Hospital, Athens, Greece; 3 Department of Radiology, National and Kapodistrian University of Athens School of Medicine, Athens, Greece

## Abstract

Periostin is an extracellular matrix protein that is implicated in the biology of normal bone remodeling and in different cancer cell growth and metastasis. However, there is no information on the role of periostin in multiple myeloma (MM). Thus, we evaluated periostin in six myeloma cell lines *in vitro*; in the bone marrow plasma and serum of 105 newly diagnosed symptomatic MM (NDMM) patients and in the serum of 23 monoclonal gammopathy of undetermined significance (MGUS), 33 smoldering MM (SMM) patients, 30 patients at the plateau phase post-first-line therapy, 30 patients at first relapse and 30 healthy controls. We found high levels of periostin in the supernatants of myeloma cell lines compared with ovarian cancer cell lines that were not influenced by the incubation with the stromal cell line HS5. In NDMM patients the bone marrow plasma periostin was almost fourfold higher compared with the serum levels of periostin and correlated with the presence of fractures and of diffuse magnetic resonance imaging pattern of marrow infiltration. Serum periostin was elevated in NDMM patients compared with healthy controls, MGUS and SMM patients and correlated with advanced disease stage, high lactate dehydrogenase, increased activin-A, increased bone resorption and reduced bone formation. Patients at first relapse had also elevated periostin compared with healthy controls, MGUS and SMM patients, while even patients at the plateau phase had elevated serum periostin compared with healthy controls. These results support an important role of periostin in the biology of myeloma and reveal periostin as a possible target for the development of antimyeloma drugs.

## Introduction

Multiple myeloma (MM) is characterized by the presence of osteolytic bone disease. Almost 80% of myeloma patients at baseline and up to 90% of patients at some stage of their disease have evidence of bone loss, leading to devastating skeletal complications. The interactions between myeloma cells and bone marrow stromal cells lead to the overproduction of several chemokines and cytokines that are responsible for this imbalance between osteoclast and osteoblast activity.^1^

Periostin, previously known as osteoblast-specific factor, is a disulfide-linked cell adhesion protein that belongs to the fasciclin family and is mainly produced by stromal cells. It is a matricellular protein, which is expressed in the periosteum after mechanical stress and is involved in bone formation and normal bone remodeling process.^2, 3^ The exact role of periostin on bone remodeling is not totally clear to date, although it is known that osteoclasts express periostin during *in vitro* differentiation, whereas the expression of periostin peaks in the early phases of osteoblast differentiation and decreases later at the start of mineralization.^4^ Novel data suggest that periostin serves both as a structural molecule of the bone matrix and a signaling molecule through integrin receptors and Wnt-β-catenin pathway, whereby it stimulates osteoblast functions and bone formation.^5^ The significance of periostin on bone biology becomes evident by its influence on bone loss in several bone disorders. High serum periostin is independently associated with increased fracture risk in postmenopausal women,^6, 7^ whereas high periostin has been associated with disease activity, systemic inflammation and bone damage in ankylosing spondylitis.^8^ Periostin expression is upregulated by several members of the transforming growth factor-β superfamily, including activin-A, which has been found to be elevated in MM by our group.^9^ Recent clinical evidence suggests that periostin stimulates metastatic growth by promoting cancer cell survival, invasion and angiogenesis in several cancers.^10, 11, 12^ Especially, bone metastases from breast cancer induce overexpression of periostin by surrounding stromal cells and serum levels of periostin are elevated in such patients.^13^ However, there is no information on the role of periostin in MM. The aim of the study was to evaluate the production of periostin by myeloma cells *in vitro* as well as serum and bone marrow plasma levels of periostin in a large number of MM patients and explore possible correlations with clinical and laboratory data, including disease stage and extent of lytic bone disease.

## Materials and methods

First, we evaluated the periostin production in the supernatant of myeloma cell lines and also by ovarian cancer cell lines. Then, we scheduled a prospective study for the measurement of periostin in myeloma patients.

### *In vitro* study design

#### Myeloma and ovarian cancer cell lines

We evaluated periostin in the supernatants of six myeloma cell lines (LR5, MR20, L363, U266, H929 and JJN3) and four ovarian cancer cell lines (A2780, C30, OVCA3 and SKOV3) before and after incubation for 24 and 48 h with stromal cell line HS5.

All myeloma and ovarian cancer cell lines A2780 and C30 were maintained in RPMI-1640 (Biosera, Bellbrook Industrial Estate, UK) supplemented with 10% fetal bovine serum (FBS; Biosera). Bone stromal cell line (HS5) were maintained in Dulbecco's modified Eagle's medium (Gibco) with 10% FBS. Ovarian cancer cells OVCA3 was maintained in RPMI-1640 with 20% FBS and SKOV3 was maintained in McCoy's 5A (modified) Medium (Gibco) supplemented with 10% FBS. All cells were propagated in standard cell culture conditions (5% CO_2_, 37 °C) in Cell Culture Treated T75 Flasks. Media were replenished every 2–3 days. Once cells had reached 80–90% confluency, cells were split (1/3) in new T75 flasks.

#### Tumor–stromal co-cultures

Cultures of all cell lines were seeded at ~6 × 10^5^ cells per well in a six-transwell plate and cocultures containing HS5 cells were plated at ~2 × 10^5^ cells (3:1 ratio) per well and maintained in standard culture conditions. Media were carefully removed after 24 and 48 h, centrifuged for 5 min at 1000 r.p.m. and the supernatant was collected for further analysis.

### Clinical study design

This was a prospective study for the evaluation of levels of periostin in the serum and bone marrow plasma of myeloma patients and their correlation with features of the disease, including overall survival.

#### Inclusion and exclusion criteria

The inclusion criteria of the study included (i) adult patients with newly diagnosed symptomatic myeloma (NDMM) before the administration of any kind of therapy; (ii) myeloma patients at the time of their first relapse (RMM); (iii) myeloma patients who are at the plateau phase of their disease for at least 6 months after the end of their frontline treatment; (iv) patients who have given their written informed consent for blood sampling and for recording of their medical data, which is pertinent to the purposes of this study.

The exclusion criteria included (i) patients <18 years; (ii) presence of autoimmune disorders or other malignant diseases; (iii) use of medication that could alter the levels of the studied parameters (that is, bisphosphonates) during the last 6 months before measurement.

#### Study end points

The primary end point of the study was the evaluation of serum levels of periostin in NDMM at the time of diagnosis and their comparison with those of patients with monoclonal gammopathy of undetermined significance (MGUS) and asymptomatic/smoldering MM (SMM).

Secondary end points included (i) measurement of bone marrow plasma periostin in NDMM; (ii) correlation of serum and bone marrow plasma periostin with disease features (stage, osteolytic disease, bone markers, lactate dehydrogenase (LDH), magnetic resonance imaging (MRI) pattern of marrow infiltration and so on); (iii) correlation of serum and bone marrow plasma periostin with survival; (iv) evaluation of circulating periostin in myeloma patients at the plateau phase of the disease after frontline therapy (at least 6 months with stable M-protein without criteria confirming progression) and in RMM patients at the time of first relapse.

#### Patients' enrollment

The enrollment period was between January 2009 and January 2011. Patients were informed about the objectives and the details of the present study before they gave their approval and signed the informed consent form. The study was conducted according to the principles defined by the 18th World Medical Association Assembly (Declaration of Helsinki, 1964) and all its future amendments. The study protocol was designed and executed according to the guidelines and regulations pertaining to studies in Greece as well as the Good Clinical Practice Guidelines as defined by the International Conference of Harmonization. The study was approved by the local ethics committee.

#### Control Groups

In this study, circulating periostin was also measured in 23 patients with MGUS and 33 patients with SMM at the time of diagnosis. MGUS and SMM patients were diagnosed during the same recruitment period and had similar age and gender with NDMM patients (Table 1). The medical history of MGUS and SMM patients was recorded to assure that they had no history of autoimmune disorder or bone disease and that they did not receive any drug that could alter the studies molecules during the last 6 months (that is bisphosphonates). Circulating periostin was also evaluated in the serum of 30, gender- and age-matched, healthy controls. Each healthy subject was examined to ensure that there was no evidence of bone disease (that is, osteoporosis, which was excluded using dual-emission X-ray absorptiometry scans of the lumbar spine and the femoral necks or osteoarthritis, which was excluded by plain radiography) and no medication that could alter the normal bone turnover during the past 6 months.

#### Data recording and quality assurance

Data were collected from the medical files of the patients. Clinical study monitor performed source data verifications and ensured the accuracy of these data. Among other data we recorded treatment data, treatment outcome according to the International Myeloma Working Group (IMWG) criteria,^14^ and patients' overall survival.

#### Measurement of periostin and of bone markers

For NDMM and RMM patients, the serum (and bone marrow plasma) was collected and stored at the time of diagnosis or relapse, respectively. For patients at the plateau phase of the disease serum was collected at the time of confirmation of the plateau (at least 6 months with stable M-protein without the IMWG criteria confirming progression). After vein-puncture serum was separated within 4 h and stored at −80 °C until the day of measurement. Similarly, bone marrow plasma was separated within 4 h after bone marrow aspiration and stored at −80 °C until the day of measurement.

Periostin was measured in the bone marrow plasma and in the serum of NDMM and in the serum of all other patients and controls using and enzyme immunoassay (USCN Life Science Inc., Wuhan, China). The method has an intra-assay coefficient of variation (CV) of <10% and an interassay CV of <12%. In all NDMM patients and controls, we also measured serum activin-A using an Enzyme-Linked Immunosorbent Assay method (R&D Systems Inc., Minneapolis, MN, USA; sensitivity 3.67 pg/ml, intra-assay CV 4.2–4.4%, interassay CV 4.7–7.9%), serum C-telopeptide of collagen type-I (CTX), which is a bone resorption marker (chemiluminescence immunoassay, CTX-I Crosslaps; ImmunoDiagnostic Systems (IDS), Herlev, Denmark; sensitivity 0.033 ng/ml, intra-assay CV 1.7–3.0%, interassay CV 2.5–10.9%) and bone-specific alkaline phosphatase (bALP), which is a bone formation marker (Ostase BAP; ImmunoDiagnostic Systems, Herlev, Denmark; sensitivity 1.0 μg/l; intra-assay CV 1.5–2.7% interassay CV 3.0–6.5%).

#### Evaluation of myeloma-related bone disease

Skeletal surveys using conventional radiography were performed at the time of diagnosis for NDMM and for RMM patients. A grading of bone morbidity into three stages according to this radiographic evaluation was made: group A included patients with no lytic lesions; group B included patients with 1–3 osteolytic lesions; and group C included patients with more than three osteolytic lesions and/or a pathological fracture due to MM. We have used the <3/>3 as cutoff for bone lesions, as advanced bone disease includes more than three lytic lesions in the Durie–Salmon staging system.

#### Evaluation of MRI pattern of marrow infiltration

T1-weighted (repetition time/echo time (TR/TE): 641/10, turbo factor 4), short-inversion recovery (TR/TE/inversion time (TI): 2000/70/150) and contrast-enhanced T1-weighted MR images (TR/TE) were obtained in symptomatic MM patients at diagnosis in the sagittal plane for the thoracic spine and for the lumbar spine and in the axial plane for the pelvis with a 1.5 T unit (Phillips Medical Systems, Eindhoven, The Netherlands). MR images were analyzed for pattern of myelomatous involvement. The pattern of marrow involvement on MR images was characterized as: (1) normal when there was no evidence of abnormal signal intensity; (2) focal, which consisted of localized areas of abnormal marrow; (3) diffuse, in which normal bone marrow signal intensity is completely absent; (4) variegated, which consists of innumerable small foci of disease on a background of intact marrow.

### Statistical analysis

Data for continuous variables are presented as mean±s.e.m. Data for categorical variables are presented as numbers and/or percentages. Kolmogorov–Smirnov test was used to test the normality of distribution of continuous variables. Paired *T*-test or Wilcoxon's signed-rank test were used to test for differences within the levels of continuous variables. Independent samples *T*-test or Mann–Whitney test were used for between-group comparisons, in cases of two groups of continuous variables. One-way analysis of variance or Kruskal–Wallis test were used in cases of more than two groups of continuous variables. In case of statistically significant difference in analysis of variance or Kruskal–Wallis test, Tukey's *post hoc* adjustment was used for multiple pairwise comparisons. *χ*^2^ or Fischer's exact test were used for between-group differences in categorical variables. Spearman's (*r*_s_) coefficient of correlation was used for bivariate correlations between continuous variables. Partial coefficient (*r*_p_) was used for binary correlations adjusted for cofounders. A two-sided *P*-value of <0.05 was considered statistically significant in all the above tests. Statistical analysis was performed by SPSS 21.0 for Macintosh (IBM Corp., Armonk, NY, USA).

## Results

### *In vitro* study

**Figure 1:**
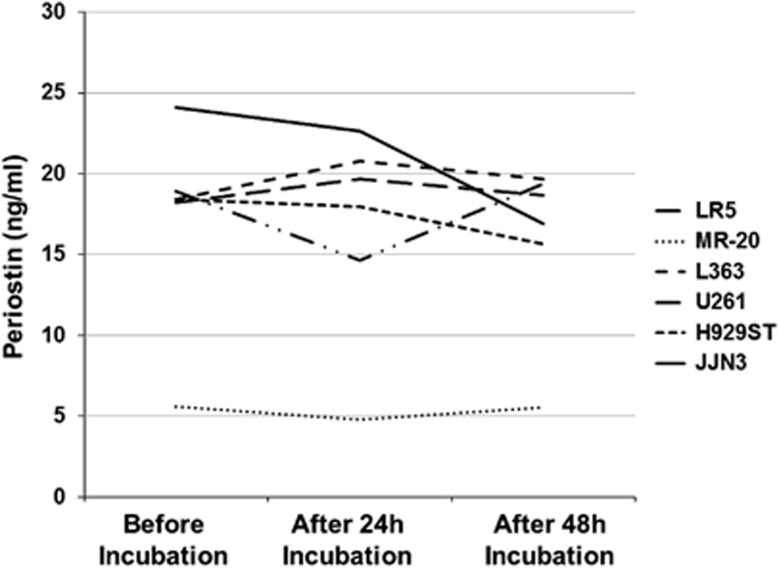
Levels of periostin in the supernatants of the six studied myeloma cell lines, before and after 24 and 48 h of incubation with the stromal HS5 cell line.

The periostin levels in the supernatants of the myeloma cell lines were higher comapred with those found in the supernatants of the ovarian cancer cell lines (mean±s.d.: 17.2±6.14 vs 2.98±1.92 ng/ml; *P*=0.001; levels of periostin in the RPMI+FBS was 0.595 ng/ml). There was no difference regarding periostin concentrations among the different myeloma cell lines or among the different ovarian cancer cell lines. After incubation for 24 and 48 h with stromal cell line HS5 (periostin level in the HS5 supernatant was 0.227 ng/ml), there was no alteration in the periostin levels of the supernatants of the myeloma cell lines (Figure 1). On the contrary, the ovarian cancer cell lines showed a marked increase in the periostin concentration after incubation with HS5 after 48 h (18.3±16.3 ng/ml, *P*=0.023) but not after 24 h (1.54±0.50 ng/ml).

### Clinical study

#### Patients

One hundred and five NDMM patients along with 30 patients at first plateau and 30 patients with RMM at first relapse participated in this study. The characteristics of these patients are depicted in Table 1. In all these patients periostin was measured in the serum, whereas in 72 NDMM, who gave the respective consent, periostin was also measured in their bone marrow plasma. Regarding bone involvement of NDMM patients, 74% had osteolytic lesions and most of them (46%) had more than three lytic lesions and/or a fracture. More specifically, 22 (21%) patients at diagnosis had a pathological fracture (20 a vertebral fracture). In MRI, 37% of patients had a focal pattern of infiltration and 30% a diffuse pattern of marrow infiltration.

#### Levels of periostin in patients and controls

**Figure 2:**
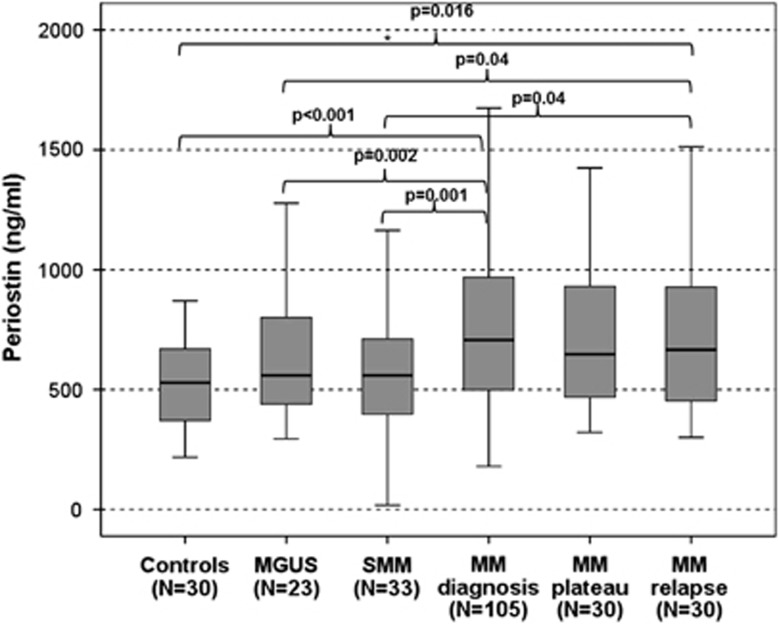
Levels of serum periostin in myeloma patients and controls.

The mean periostin levels of the bone marrow plasma of the 72 NDMM patients were 3406 ng/ml (±5320 ng/ml), almost fourfold higher compared with the respective values of serum periostin of symptomatic MM patients at diagnosis (911±694 ng/ml). Serum periostin of NDMM patients were increased compared with healthy controls (537±190 ng/ml; *P*<0.001), with SMM patients at diagnosis (601±351 ng/ml; *P*=0.001) and with MGUS patients (633±271 ng/ml; *P*=0.002; Figure 2). In the 72 patients with measurements in both bone marrow plasma and serum, there was a weak correlation between the two values (*r*=0.225, *P*=0.05).

Patients with MM at the plateau phase had a borderline reduction of serum periostin concentrations (729±360 ng/ml) compared with symptomatic MM patients at diagnosis (*P*=0.05) but they continued to have increased levels compared with healthy controls (*P*=0.013). Patients with relapsed MM had also increased circulating periostin (938±847 ng/ml) compared with controls (*P*=0.016), MGUS (*P*=0.04) and SMM patients (*P*=0.04); serum periostin levels did not differ between RMM and NDMM patients.

#### Correlations among periostin and disease characteristics in NDMM patients

**Figure 3:**
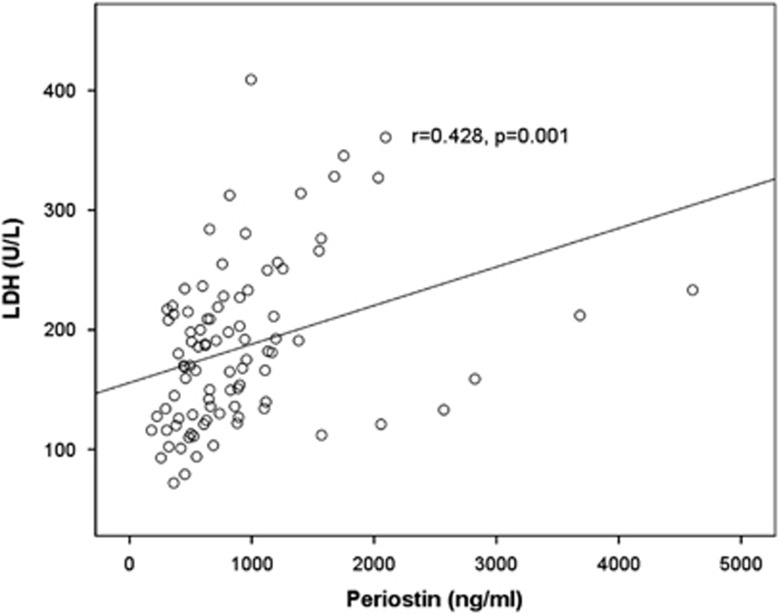
Serum levels of periostin strongly correlate with serum LDH (a marker of advanced disease).

**Figure 4:**
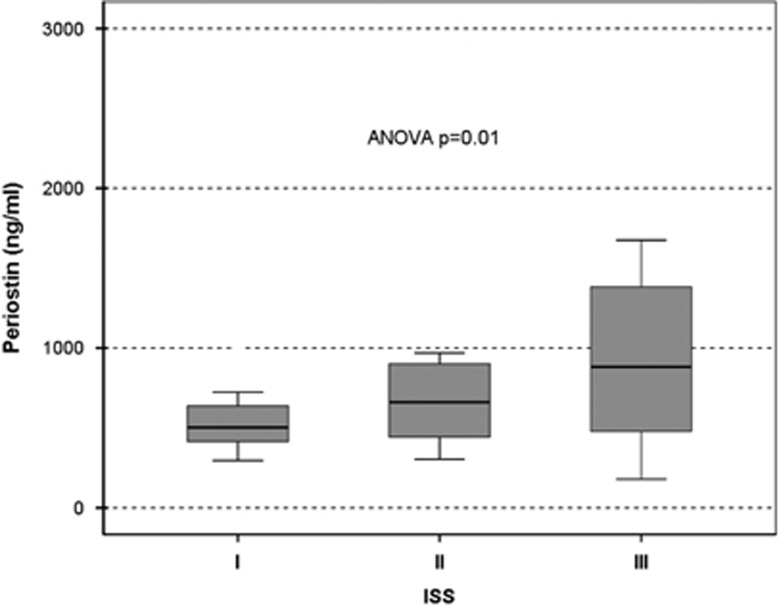
Serum levels of periostin correlate with myeloma stage (ISS).

In symptomatic NDMM patients, serum periostin strongly correlated with β2-microglobulin (*r*=0.384, *P*=0.004), LDH (*r*=0.428, *P*=0.001; Figure 3) and ISS stage (mean±s.d. of periostin values for ISS-1, ISS-2 and ISS-3 were: 542±196, 775±500 and 1036±801 ng/ml, respectively; *P*=0.01, Figure 4).

**Figure 5:**
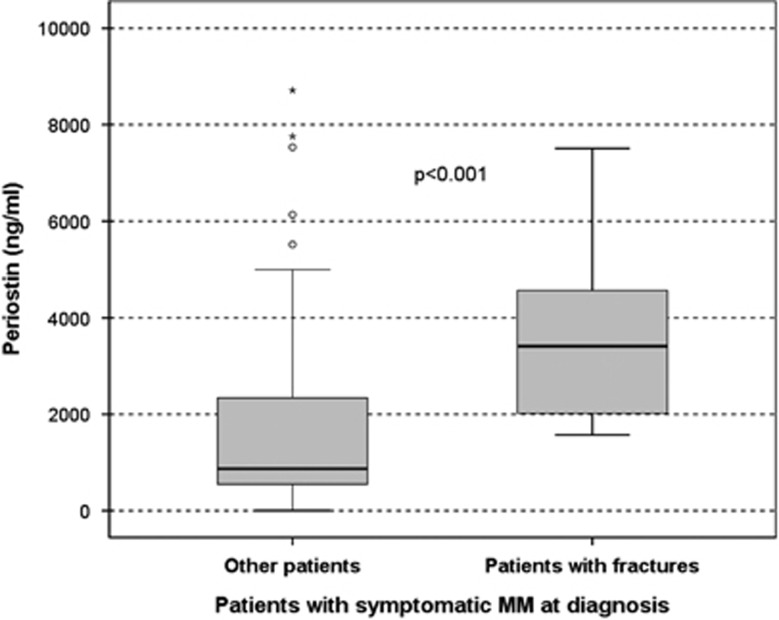
Bone marrow plasma levels of periostin are highly elevated in newly diagnosed patients with symptomatic MM and pathological fractures.

Periostin in both the marrow plasma and the serum were markedly elevated in patients with a pathological fracture at diagnosis compared with all others. More specifically, patients with a pathological fracture (*n*=22) had very high levels of marrow periostin (4944±2677 ng/ml) vs all other (2016±5899 ng/ml; *P*<0.001; Figure 5), whereas the respective values in the serum were also elevated but of lower significance (1318±907 vs 801±611 ng/ml; *P*=0.032). Bone marrow plasma periostin showed a borderline increase in patients with osteolytic lesions vs those without osteolytic lesions (3825±4118 vs 2790±3228 ng/ml; *P*=0.028), whereas serum periostin was not different among patients with or without osteolytic lesions. Furthermore, the levels of bone marrow periostin were elevated in patients with diffuse MRI pattern compared with all others (5352±7221 vs 3252±4943 ng/ml, *P*<0.05). No other correlations were observed among studied groups for periostin levels.

#### Correlations between periostin and bone markers in newly diagnosed MM patients

In symptomatic MM patients at diagnosis, serum CTX and activin-A were elevated compared with MGUS, SMM and healthy controls (*P<*0.01 for all comparisons; see Table 2). Furthermore, bALP was marginally reduced compared with MGUS, SMM and control values (*P<*0.05 for all comparisons). Serum periostin negatively correlated with bALP (*r*=−0.464, *P<*0.001), whereas marrow plasma levels positively correlated with activin-A (*r*=0.478, *P<*0.001). High circulating periostin also correlated with increased bone resorption, as assessed by CTX levels (*r*=0.369, *P*=0.005) in symptomatic patients at diagnosis.

## Discussion

In this study, we show for the first time that periostin is elevated in the serum and bone marrow plasma of patients with NDMM and correlates with advanced disease features, such as ISS-3, diffuse MRI pattern of marrow infiltration and LDH at diagnosis. Furthermore, we found higher periostin levels in the supernatants of six myeloma cell lines in comparison with four ovarian cancer cell lines. Of interest, the levels of periostin did not increase after incubation of myeloma cell lines with the stromal cell line HS5 after 24 or 48 h, suggesting that the production of periostin by the myeloma cell lines is not dependent on the stromal cells. It has been recently published that extracellular matrix proteins, including periostin, are progressively upregulated in MGUS and MM patients.^15^ In our study, circulating periostin was elevated in both NDMM and RMM at first relapse compared with MGUS patients, and also compared with SMM and healthy controls, in accordance with the above finding.

Periostin is overexpressed at the periosteal surface, and also in other collagen-rich tissues subjected to mechanical strain such as periodontal ligaments, heart valves and tendons.^5, 16^ Periostin seems to be an important mediator of the effects of mechanical factors and parathyroid hormone on cortical bone mineral density and bone strength by modulating the canonical Wnt signaling pathway with a downregulation of sclerostin expression.^17^ In our study, elevated periostin correlated with reduced bone formation as assessed by the negative correlation with a sensitive bone formation marker, bALP. Moreover, its strong positive correlation with the bone resorption marker CTX supports a role for periostin in the development of bone loss in MM. Indeed, NDMM patients with advanced osteolytic bone disease and specifically with pathological fractures at diagnosis had very high bone marrow plasma periostin compared with all others. Even periostin in the serum was elevated in NDMM patients with pathological fractures. This result is in accordance with data that high serum periostin levels are independently associated with increased fracture risk in postmenopausal women.^7, 8^ Moreover, periostin is elevated in the serum of patients with different subtypes of hemoglobinopathies, disorders that also correlate with bone loss.^18^ It is of interest that NDMM patients with fractures had markedly elevated bone marrow plasma periostin even compared with patients with high number of osteolytic lesions. One possible explanation is that periostin also correlates with bone repair that it is probably more pronounced in patients with fractures compared with all other patients. In postmenopausal status, there was no difference in circulating periostin levels between women with low and normal bone mass,^19^ whereas periostin could predict for fractures^7^ or it was elevated in patients with fractures.^8^ Furthermore, the presence of different periostin isoforms with unknown activity on bone remodeling may also explain these differences.^5^ In this study, we also found a correlation between periostin and activin-A serum levels. Activin-A is a transforming growth factor-β superfamily member that is also implicated in the biology of myeloma-related bone disease.^9, 20^ Transforming growth factor-β pathway proteins, including activin-A, upregulate periostin expression.^21^ The correlation of activin-A with periostin in our study suggests a possible combining role of the two molecules in the development of bone loss in myeloma patients.

In our study, periostin correlated with advanced disease features, including ISS-3, high LDH and diffuse pattern of marrow infiltration in MRI. Periostin has a major role in carcinogenesis and tumor progression in a number of malignancies besides its role in bone metabolism. It augments lymph angiogenesis in head and neck cancer, significantly influencing the stage of the tumor,^22^ whereas high expression of periostin correlates with poor prognosis in osteosarcoma.^12^ Similarly, increased periostin expression is seen in nearly one-third of breast carcinomas. These patients tend to show a poor clinical outcome and poor disease-specific survival.^23, 24^ Furthermore, periostin was detectable in the serum of early breast cancer patients before surgery and increased baseline serum levels predicted worse long-term survival outcomes in specific subgroups of patients.^25^ Similarly, upregulation of periostin is seen in non-small-cell lung and prostate cancer and correlates with disease stage.^26, 27^ Our report is the first, according to our knowledge, which shows high levels of periostin in myeloma patients that correlate with adverse disease features. Even patients at the plateau phase of the myeloma had elevated periostin levels in their serum compared with healthy controls. These results support a role of periostin in the biology of the disease, which is further strengthened by the observation that periostin gene is upregulated during the evolution from MGUS to overt MM.

In summary, our data suggest that periostin is elevated in the bone marrow plasma and in the serum of NDMM patients and correlates with bone fractures, extensive osteolysis and advanced disease characteristics. Serum periostin is also elevated in myeloma patients at first relapse and in patients at the plateau phase of the disease after frontline therapy. These data support an important role of periostin into the biology of MM as it has been shown in other malignancies. These results also reveal periostin as a possible therapeutic target. Recently, a periostin-neutralizing antibody, PN1-Ab, has been developed, and it attenuates tumor growth of the primary breast carcinoma in animal models.^28^ Studies in myeloma are highly anticipated.

## Figures and Tables

**Table 1:** Characteristics of patients and controls

	*MGUS*	*SMM*	*MM at diagnosis*	*MM at plateau*	*MM at first relapse*	*Controls*
No	23	33	105	30	30	30
Gender (M/F)	17/6	18/15	55/50	21/9	18/12	16/14
Median age (range)	70 (43–85)	69 (46–81)	71 (41–88)	71 (50–85)	72 (53–80)	70 (50–79)
Ig subtype: IgG/IgA/light chain only/IgD/non-secretory	13/9/0/1/0	17/12/4/0/0	65/25/10/1/4	15/8/5/0/2	16/9/4/0/1	
						
*ISS stage*						
ISS-1			29 (27.6%)	9 (30%)	8 (26.6%)	
ISS-2			34 (33.3%)	11 (36.6%)	12 (40%)	
ISS-3			42 (40%)	10 (33.3%)	10 (33.3%)	
Hb<10 g/dl			42 (40%)	5 (16.6%)	27 (90%)	
Creatinine >UNL			29 (27.6%)	1 (3.3%)	11 (36.6%)	
Creatinine ⩾2 mg/dl			17 (16.1%)	0	8 (26.6%)	
LDH>240 U/l			13 (12.3%)	0	9 (30%)	
Presence of lytic lesions			78 (74.2%)	23 (76.6%)	24 (80%)	
						
*Bone disease*						
A			27 (25.7%)	7 (23.3%)	6 (20%)	
B			30 (28.5%)	3 (10%)	2 (6.6%)	
C			48 (45.7%)	20 (66.6%)	22 (73.3%)	
						
*MRI pattern*						
Focal			39 (37.1%)			
Diffuse			32 (30.4%)			
Variegated			5 (4.7%)			
Normal			29 (27.6%)			

Abbreviations: Hb, hemoglobin; Ig, immunoglobulin; ISS, International Staging System; LDH, lactate dehydrogenase; MGUS, monoclonal gammopathy of undetermined significance; MM, multiple myeloma; MRI, magnetic resonance imaging; SMM, smoldering MM; UNL, upper-normal limit.

**Table 2:** Values of serum bone metabolism parameters and activin-A in all study groups

	*CTX (ng/ml)*	*bALP (U/l)*	*Activin-A*
NDMM (*N*=105)	0.75±0.31 (0.517)	12.1±1.8 (11.6)	1228±789 (555)
Healthy controls (*N*=30)	0.24±0.23 (0.180)	14.3±9.6 (13.1)	355±112 (321)
SMM (*N*=33)	0.47±1.05 (0.253)	15.8±9.2 (14.7)	412±223 (347)
MGUS (*N*=23)	0.37±0.17 (0.296)	19.2±11.3 (17.9)	471± 332 (351)
*P*-value (NDMM vs controls)	<0.001	0.045	<0.001
*P*-value (NDMM vs SMM)	0.009	0.032	<0.001
*P*-value (NDMM vs MGUS)	0.007	0.018	0.008
*P*-value (SMM vs controls)	0.178	0.187	0.678
*P*-value (SMM vs MGUS)	0.564	0.026	0.876
*P*-value (MGUS vs controls)	0.106	0.012	0.690

Abbreviations: bALP, bone-specific alkaline phosphatase; CTX, C-telopeptide of collagen type-I; MGUS, monoclonal gammopathy of undetermined significance; MM, multiple myeloma; NDMM, newly diagnosed symptomatic MM; SMM, smoldering MM.

Mean±s.d. (median).
